# Using Quantitative Imaging for Personalized Medicine in Pancreatic Cancer: A Review of Radiomics and Deep Learning Applications

**DOI:** 10.3390/cancers14071654

**Published:** 2022-03-24

**Authors:** Kiersten Preuss, Nate Thach, Xiaoying Liang, Michael Baine, Justin Chen, Chi Zhang, Huijing Du, Hongfeng Yu, Chi Lin, Michael A. Hollingsworth, Dandan Zheng

**Affiliations:** 1 Department of Radiation Oncology, University of Nebraska Medical Center, Omaha, NE 68198, USA; kpreuss@huskers.unl.edu (K.P.); nate.thach@huskers.unl.edu (N.T.); mbaine@unmc.edu (M.B.); jschen1@stu.naperville203.org (J.C.); clin@unmc.edu (C.L.); 2 Department of Nutrition and Health Sciences, University of Nebraska Lincoln, Lincoln, NE 68588, USA; 3 Department of Computer Science, University of Nebraska Lincoln, Lincoln, NE 68588, USA; hfyu@unl.edu; 4 Department of Radiation Oncology, Mayo Clinic, Jacksonville, FL 32224, USA; liang.xiaoying@mayo.edu; 5 Naperville North High School, Naperville, IL 60563, USA; 6 School of Biological Sciences, University of Nebraska Lincoln, Lincoln, NE 68588, USA; zhang.chi@unl.edu; 7 Department of Mathematics, University of Nebraska Lincoln, Lincoln, NE 68588, USA; hdu@unl.edu; 8 Eppley Institute for Research in Cancer, University of Nebraska Medical Center, Omaha, NE 68198, USA; mahollin@unmc.edu; 9 Department of Radiation Oncology, University of Rochester, Rochester, NY 14626, USA

**Keywords:** radiomics, quantitative imaging, pancreatic cancer, machine learning, deep learning

## Abstract

**Simple Summary:**

With a five-year survival rate of only 3% for the majority of patients, pancreatic cancer is a global healthcare challenge. Radiomics and deep learning, two novel quantitative imaging methods that treat medical images as minable data instead of just pictures, have shown promise in advancing personalized management of pancreatic cancer through diagnosing precursor diseases, early detection, accurate diagnosis, and treatment personalization. Radiomics and deep learning methods aim to collect hidden information in medical images that is missed by conventional radiology practices through expanding the data search and comparing information across different patients. Both methods have been studied and applied in pancreatic cancer. In this review, we focus on the current progress of these two methods in pancreatic cancer and provide a comprehensive narrative review on the topic. With better regulation, enhanced workflow, and larger prospective patient datasets, radiomics and deep learning methods could show real hope in the battle against pancreatic cancer through personalized precision medicine.

**Abstract:**

As the most lethal major cancer, pancreatic cancer is a global healthcare challenge. Personalized medicine utilizing cutting-edge multi-omics data holds potential for major breakthroughs in tackling this critical problem. Radiomics and deep learning, two trendy quantitative imaging methods that take advantage of data science and modern medical imaging, have shown increasing promise in advancing the precision management of pancreatic cancer via diagnosing of precursor diseases, early detection, accurate diagnosis, and treatment personalization and optimization. Radiomics employs manually-crafted features, while deep learning applies computer-generated automatic features. These two methods aim to mine hidden information in medical images that is missed by conventional radiology and gain insights by systematically comparing the quantitative image information across different patients in order to characterize unique imaging phenotypes. Both methods have been studied and applied in various pancreatic cancer clinical applications. In this review, we begin with an introduction to the clinical problems and the technology. After providing technical overviews of the two methods, this review focuses on the current progress of clinical applications in precancerous lesion diagnosis, pancreatic cancer detection and diagnosis, prognosis prediction, treatment stratification, and radiogenomics. The limitations of current studies and methods are discussed, along with future directions. With better standardization and optimization of the workflow from image acquisition to analysis and with larger and especially prospective high-quality datasets, radiomics and deep learning methods could show real hope in the battle against pancreatic cancer through big data-based high-precision personalization.

## 1. Introduction

Pancreatic cancer remains an unsolved global healthcare problem, has the highest mortality rate of all major cancers, and is expected to take the lives of more than 49,830 people in the US in 2022 alone [1]. While the five-year survival rate has risen considerably for many other cancers over the past century, it has remained rather stagnant for pancreatic cancer despite intense healthcare efforts, staying in the single digits for decades and only recently rising to 10.8% [1]. By the time of diagnosis over half of pancreatic cancers are metastasized, and for these patients the five-year survival rate is only 3% [1]. The dire disease situation reflects our inability to diagnose pancreatic cancer early and to effectively treat it. Our failure to diagnose the disease early results in part from the inaccessibility of the organ, difficulties in detecting small pancreatic lesions by conventional imaging approaches, and a poor understanding of the spectrum of heterogeneity in pancreatic cancer. The extremely high degree of heterogeneity in this disease underscores the challenge of effectively treating any given patient while balancing tumoricidal effects and normal tissue toxicity. Personalized medicine has hence been proposed for this most fatal cancer through a systems approach that integrates multi-omics data to tailor medical treatment to the individual characteristics of each patient.

Despite its high death rate, pancreatic cancer has long been considered unsuitable for screening programs [2]. Major reasons for this include the absence of clinical symptoms in the early stage, the variable risks of malignant transformation associated with different precancerous pancreatic diseases and the difficulties in clinical and imaging-based differential diagnosis, the rare incidence of malignant diseases, and the high risk of pancreatic biopsy and surgery [3]. Similarly, the same challenges remain in the differential diagnosis of pancreatic cancer versus benign diseases as well as among different types of pancreatic cancer. Furthermore, reliable biomarkers are lacking to effectively stratify patients based on prognosis risk and predicted treatment response. Together with pancreatic cancer remaining the most fatal cancer and screening programs being not widely used, research on early detection, diagnosis, risk stratification, prognosis prediction, and treatment optimization remains necessary in order to move towards personalized medicine in pancreatic cancer.

Medical imaging plays an important role in all these crucial effort directions. Various imaging modalities are already routinely used for cancer screening, detection and diagnosis, treatment planning, and long-term follow up. Due to the inaccessibility of the tumor and the risks associated with invasive procedures, pancreatic cancer is especially reliant on imaging. Moreover, imaging is naturally poised to assess disease heterogeneity and progression, two important traits of pancreatic cancer. Quantitative imaging, a recently new area of study based on medical imaging, helps to solve these problems. By approaching images as data able to be mined, instead of merely pictures in conventional radiology, quantitative imaging allows for further information to be extracted from medical images as well as for global assessments across large patient populations. Therefore, these new quantitative approaches hold the promise of detecting pancreatic cancer characteristics that the naked eye alone cannot perceive from conventional medical imaging, opening new doors for personalized medicine in pancreatic cancer.

**Figure 1:**
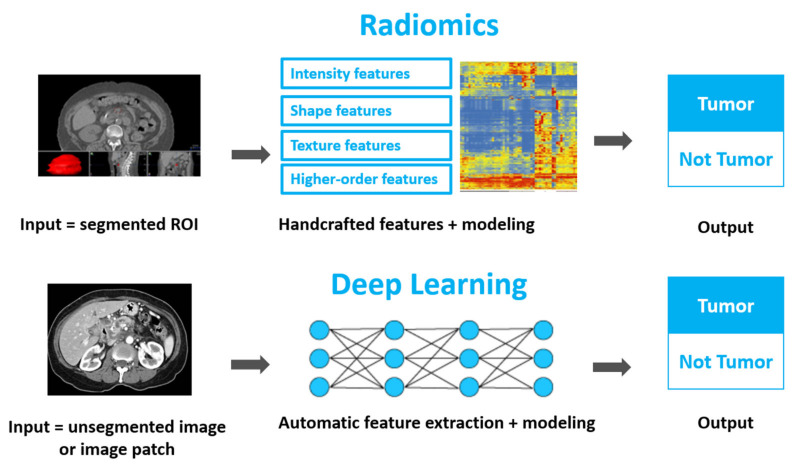
A schematic drawing comparing the radiomics approach and the deep learning approach, using an example case of tumor detection.

To date, two main types of quantitative imaging approaches have been researched for all kinds of medical problems: radiomics and deep learning. Radiomics involves extraction of hand-crafted features (such as shape, intensity, texture, and wavelet) from imaging, usually based on segmented regions of interest [4]. Deep learning is a subfield of machine learning involving algorithms inspired by the structure and function of the brain or neural networks [5]. While it can be used as an analysis method for any type of data, including radiomics, in the narrow sense referred to in this paper deep learning refers to the application of deep learning methods directly on unsegmented images or image patches. Figure 1 provides a schematic illustration to contrast the two approaches. While both approaches take advantage of computational methods to decipher encoded high-dimensional information from medical images that is conventionally unfathomable, they each have their strengths and challenges. Newer studies sometimes combine radiomics and deep learning approaches, thereby yielding superior results.

This review provides a systematic review of radiomics and deep learning applications pertaining to pancreatic cancer. The literature search was conducted using the PubMed (https://pubmed.ncbi.nlm.nih.gov/, accessed on 8 July 2021) and ScienceDirect databases (https://www.sciencedirect.com/, accessed on 8 July 2021). The keywords “pancreatic cancer”, “pancreas”, “radiomics”, “imaging”, “machine learning”, and “deep learning” were used to filter relevant full articles for reference for studies published until November 2021 at the writing of this review. While there have been several reviews of pancreatic radiomics and machine learning, existing reviews have focused on either the history of the field, certain biological perspectives, or a specific method or application. Therefore, this paper hopes to fill a gap by providing a comprehensive review of pancreatic cancer radiomics and deep learning from both the clinical and the technical perspective in order to be of use to medical professionals as well as relevant researchers.

## 2. Technical Overview: Quantitative Imaging and Two Analytical Approaches

As with other types of pictures in our everyday life, medical images have undergone an analog-to-digital evolution during the past decades. New imaging modalities have been invented, and visualization of living systems has expanded from two-dimensional (2D images such as X-rays) to 3D (computed tomography or CT) and 4D (using time as the fourth dimension to image cyclic motion) as well as from mere anatomy to physiology (such as positron emission tomography, or PET, and functional magnetic resonance imaging, MRI). With technological advancements and new modalities has come a growing ability to generate medical images with far greater detail, accuracy, and precision. It is on these foundations, the medical community began to perceive medical images as quantitative and minable data rather than merely as pictures. New approaches aim to automatically generate quantitative knowledge that is often qualitative/descriptive and manual from conventional radiology and to exploit the expanded depth and breadth of information embedded within advanced medical images which cannot be discovered by the naked eye. In addition to the visualization they provide, medical images can enable quantitative analyses, much like other laboratory tests, with valuable information connected both to molecular-level biological phenotypes and macroscopic clinical presentations. Advantageously, these new approaches harness data from medical imaging already produced in the clinical workflow, providing a cost-effective yet powerful data source. Moreover, as these quantitative approaches are based on medical imaging, they are perfect for globally assessing the heterogeneity of a disease as well as longitudinally monitoring disease progression.

Quantitative medical image analysis usually takes one of two approaches, as illustrated in Figure 1. Extracting handcrafted features from images is the radiomics approach, and usually involves segmenting a region of interest (ROI) and calculating hundreds to thousands of predefined mathematical constructs, called radiomic features [6]. The radiomics workflow typically consists of several steps: image acquisition, ROI segmentation, feature extraction, and model building [6]. The second approach, deep learning, refers herein to those artificial intelligent methods that implicitly analyze medical images and hence do not rely on the initial expert inputs of ROI segmentation and handcrafted features. It should be pointed out that in this paper we address other types of machine learning methods, such as reinforcement learning, in the deep learning category; this distinction is based on the common definition of learning from the image instead of from extracted features.

**Figure 2:**
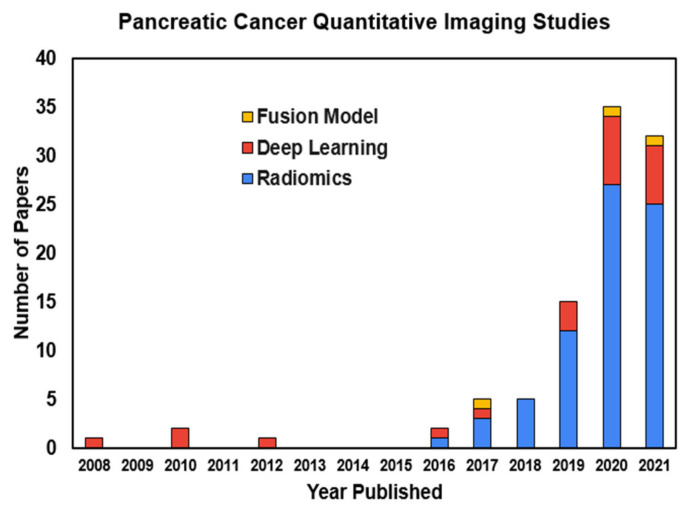
Quantitative imaging studies using radiomics and deep learning methods in pancreatic cancer-related research by year of publication.

Sometimes, the two approaches can be combined to create “fusion” models. When employed, such fusion models often outperform single-method approaches. Figure 2 plots a histogram of pancreatic cancer-related papers using these quantitative imaging approaches that have been published in different years. A growing trend can be seen, especially during the most recent several years.

### 2.1. Technical Basis: Radiomics

**Figure 3:**
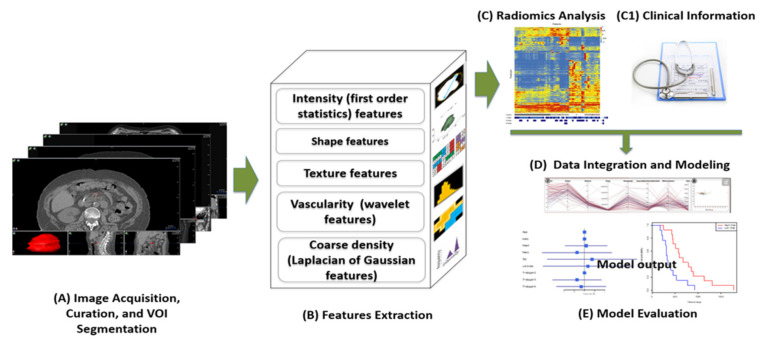
In a typical radiomics workflow, medical images are acquired and curated and volumes of interest (VOIs) such as pancreatic tumors are segmented (**A**). From the segmented VOI images, hundreds to thousands of radiomic features are then be extracted (**B**). After conducting preliminary radiomics analysis such as feature selection (**C**) and possibly adding clinical and biological information (C1), all features can be integrated through advanced statistical and/or machine learning methods to develop predictive models (**D**). The model accuracy and robustness can then be evaluated on validation and testing datasets (**E**).

The term radiomics was coined from other “omics” terms such as genomics and proteomics by combining the words “radiology” and “omics”. Simply put, radiomics is the branch of high-throughput data mining research in radiology that involves extracting an array of hand-crafted quantitative features from medical images. As mentioned above, a typical radiomics workflow consists of several basic steps, which are depicted in Figure 3.

For pancreatic cancer, typical image modalities include those used in clinical management of pancreatic diseases such as CT, MRI, PET, and endoscopic ultrasound (EUS). Among these, CT is the most widely used imaging modality for pancreatic cancer owing to its high spatial and temporal resolution, benchmarking sensitivity, and specificity in pancreatic diseases, as well as its lower cost and wider availability compared with MRI and PET. A bi-phase or tri-phase pancreatic protocol is usually used with an iodinated contrast agent for CT image acquisition. With its good soft tissue contrast, MRI is increasingly used to complement CT for pancreatic cancer diagnosis and management. MR cholangiopancreatography is used as a non-invasive alternative to endoscopic retrograde cholangiopancreatography. PET commonly uses fluorine 18-fluorodeoxyglucose (FDG), a glucose analogue, to image high-metabolic cancer. Other tracers can be employed in PET to image other biological information. PET has been used for pancreatic cancer diagnosis as well as post-therapy monitoring. Ultrasound is most often used in the EUS setting to visualize the pancreas from the duodenum or stomach and detect small focal lesions, as well as to guide biopsies.

While radiomics studies are most often conducted using one of the above-mentioned imaging modalities, they can combine two or more modalities to provide complementary and more comprehensive information. From the obtained image, one or more ROIs are delineated or segmented to allow subsequent analysis to focus on the ROIs. For pancreatic cancer-based radiomics the ROI is usually the pancreatic tumor, or occasionally metastatic pancreatic cancer lesions. For pancreatic cancer detection, the ROI can be the entire pancreas or sub-regions thought to potentially contain the cancer. Segmentation can be carried out either manually, semi-automatically, or automatically. Automatic segmentation is desirable because it automates a labor-intensive step and is therefore an essential factor for securing the large amount of data required for high-quality quantitative imaging studies. Numerous computer algorithms have been developed for automatic segmentation, from simple thresholding to atlas-based methods and artificial intelligence-based algorithms. Human interactions, such as setting the algorithm’s start point, may be required, making it a semi-automatic process. Automatic and semi-automatic segmentation methods are known to save labor and improve workflow efficiency and interpatient segmentation consistency [7]. However, when compared with most other types of cancers pancreatic tumor and pancreas anatomical segmentation can be quite difficult due to the lack of contrast in boundaries and due to heterogeneity both within the ROIs and in the background. Therefore, automatic/semi-automatic segmentation methods for pancreatic cancer are an active area of research and development, and manual segmentation remains the mainstay in pancreatic cancer radiomics [7]. The only exception is in PET-based studies, where automatic segmentation can be employed based on the thresholding of standardized uptake values (SUVs). It is worth noting that despite the longer time required to delineate pancreatic tumors compared to other cancers such as lung tumors, manual segmentation of pancreatic cancer suffers higher interobserver variabilities as well [8,9]; this can lead to higher segmentation uncertainties which are then propagated through the radiomics workflow. To somewhat mitigate this, studies have used multiple observers to enhance the robustness of manual segmentation; however, there is no standard practice regarding this. Existing studies showed largely varying degrees of attention to segmentation, with several taking into consideration inter- and intra-observer reproducibility, others using a single observer, and others reporting no details about segmentation.

Radiomic features are mathematically defined quantities computed from an image ROI. They can be divided into different categories, such as intensity, shape, texture, and higher-order features [6]. Intensity features are sometimes called first-order statistical features. They are histogram-based quantities such as minimum, mean, median, and maximum. Other examples include skewness, which reports the asymmetry of the intensity distribution, kurtosis, which reports the “tailedness” versus the flatness of the distribution, and others. Shape features help to identify ROI shape and size. In addition to the volume and maximum diameter often used in conventional radiology, shape features include various others used to quantitatively describe the ROI shape, such as sphericity, convexity, and irregularity. Texture features are usually second-order statistical features that can be calculated from various matrices depicting the inter-voxel statistical relationships between neighboring voxels. The most common texture matrices include the gray-level co-occurrence matrix (GLCM) that calculates the incidence of voxels with the same intensities at a specific distance along a specific direction and the gray-level run-length matrix (GLRLM), which calculates the number of consecutive voxels with the same intensity along a specific direction, as well as others. The texture features are especially useful for quantifying tumor heterogeneity, which is often missed or ambiguous in conventional radiology. While the above categories of radiomic features can be calculated from the original image, they can be calculated from the derived image as well after applying image filters or mathematical transformations to the original image. The latter are called higher-order features. The filters used to extract higher-order features are usually those used in typical image processing, serving a particular purpose such as highlighting details and suppressing noise. Common filters used in radiomics include wavelet, Laplacian of Gaussian, and more. With different image filters and filter parameter settings, the number of radiomic features can quickly go from a few dozens or hundreds to several thousand. It is worth noting that unlike intensity and texture features, shape features are invariant with image filters.

For radiomics modeling, the process usually involves feature selection as well as model development and validation. Because radiomics models are developed from a pool of hundreds to thousands of radiomic features (which are redundant) and the number of subjects is usually on the order of a few dozens to a few hundreds, model overfitting can become a major issue without an effective and robust feature selection or dimension reduction process. Overfitting tends to happen when a large number of features are used to model a dataset of limited size in which the model learns more from the noise in the dataset than from the signal, leading to a poor fit with new datasets. Various statistical and machine learning methods are used for radiomics dimension reduction, including minimum redundancy and maximum relevancy (mRMR), mutual information maximization, least absolute shrinkage and selection operator (LASSO), and random forest [5,6]. Through dimension reduction and feature selection a much smaller number of most relevant radiomic features can be selected, usually under a dozen. When a few radiomic features are determined to be the most significant and useful for the prediction in question, they are often called a radiomic signature. Radiomic signatures are used for model development with both simple statistical and more complex machine learning methods. A Cox model is one example of a statistical model that is frequently used, simple, and robust [10]. Other common methods include naïve Bayesian, support vector machine, neural network, random forest, and more [10]. If the predicted outcome is discrete, and especially if it is dichotomic (i.e., positive vs. negative), a classification model is built, whereas if it is continuous, a regression model is used. To develop a more robust and generalizable radiomic model, proper model validation and testing is often necessary. A good way to do this is to divide the dataset into a training dataset for training the model and tuning model parameters and a validation dataset for confirming model validity; one or more external independent datasets can then be used for model testing to further confirm the validity and the generalizability of the developed model. Datasets for pancreatic cancer are often more limited than other types of cancers due to the relative rarity and rapid progression of the disease. Where external datasets or large dataset sizes are lacking, methods such as cross-validation and bootstrapping have been used to maximize data usage and mitigate overfitting [6].

### 2.2. Technical Basis: Deep Learning

Machine learning is an important branch of artificial intelligence and a powerful alternative to statistical methods for data analysis and model building. Unlike statistical modeling, which relies on theories, or conventional computer science, which relies on explicit programming, machine learning involves computers learning from data and performing tasks such as model building without being explicitly programmed to. Conventional computer algorithms often rely on explicitly programmed “if–then” logics, while machine learning learns from data without rules-based programming. Traditional machine learning algorithms use rather simple structures such as linear regression and decision trees [10]. In contrast, deep learning, a branch of machine learning that takes this a step further and completely removes the human component, uses more complex algorithms inspired by the structure and function of human brains [11]. These algorithms are often called “neural networks” because they mimic the structure and information relay between neurons in a human brain, and different types of neural networks differ in how information flows through individual “neuron” layers [11]. In the case of quantitative imaging for personalized pancreatic cancer, neural networks all have an input, the pancreas image, and an output, the image-based prediction. The prediction can be a classification problem, such as whether a lesion is benign or malignant or, what histology a lesion belongs to, or a regression problem, such as predicting the expected survival time of a patient. As described in the above section, machine learning or even deep learning approaches can be applied to analyze radiomics data; the key difference between radiomics and deep learning is that deep learning as referred to in this review takes the image as its direct input. In contrast, feature extraction takes place first in radiomics, and the inputs for subsequent data analysis are these handcrafted imaging features.

The convolutional neural network (CNN) is currently the most commonly used deep learning method for medical imaging-based studies [10]. Deep learning algorithms automate the process of feature extraction by inherently learning the important features and applying them to map the input to the output. In a classical artificial neural network (ANN), this is realized by processing the input through hidden layers which learn the weights of different nodes (neurons) and apply appropriate activation functions to yield nonlinearity for learning the complex relationship between the input and output. There are several challenges using ANNs for image-based deep learning problems. Because the 2D or 3D image in ANNs is first converted to a 1D vector, the number of nodes drastically scales up with the image size, making it very computationally expensive. In addition, the spatial information in the input image is lost in this linear conversion. In contrast, CNNs apply filters (called “kernels”, hence the term “convolution” in CNN) to flow the information rather than the 1D conversion used in ANNs. For each layer of kernel convolution, there is a subsequent activation step to introduce nonlinearity into the network and a pooling step to reduce the dimensionality of the feature map while preserving critical feature information. CNNs are therefore well-suited for image-based deep learning because of their ability to capture spatial features from an image and extract relevant features at a low computational cost.

Similar to the radiomics workflow, a good deep learning study will be designed to have a training dataset, validation dataset, and test dataset. An advantage of deep learning over radiomics is that it no longer requires the ROI segmentation step. This saves substantial time and effort requirements, as segmentation is a labor-intensive step of radiomics, and avoids propagation of segmentation uncertainty into the downstream steps. Due to the complex image intensity both within the pancreas and in its background, this is especially important. On the other hand, without using domain-guided ROI or handcrafted features, the dataset size required to train a robust deep learning model is higher than for a radiomics study. In medical studies, the dataset size is usually limited; for pancreatic cancer this is even more of an issue, because rapid disease progression and typically poor outcomes further limit available data. A few different approaches are used in deep learning to mitigate the data size issue. CT, MRI, and PET images of the pancreas are 3D images; however, 2D image slices or even “patches” of 2D slices (subsections of the 2D image) are usually used as the network inputs instead of the 3D images. As each 3D image set can yield dozens to hundreds of 2D images and hundreds to thousands of 2D image patches, the data size increases dramatically, and even the much smaller image or patch drastically reduces the trainable parameters of the network. Currently, 2D CNNs are the method of choice for CNN-based deep learning approaches for pancreatic cancer. On the other hand, this approach may miss the potentially relevant 3D spatial context, which on occasion motivates investigators to employ a 3D or a 2.5D CNN architecture. Another important method for mitigating data size limitations for deep learning is data augmentation, in which operations such as flipping, rotation, translation, and scaling are used to synthesize modified data from the original data in order to increase the training dataset size. Another method is transfer learning, where standard architectures designed based on natural images with pretrained weights, such as ImageNet, are fine-tuned with specific medical images [12,13]. Despite making a certain intuitive sense, methods such as data augmentation and transfer learning may not always improve deep learning model performance.

## 3. Clinical Applications

### 3.1. Pre-Cancerous Pancreatic Lesion Diagnosis

The extreme aggressiveness of pancreatic cancer greatly dampens survival probability when the cancer is diagnosed in late stages, with a five-year survival rate of 3% for metastatic disease [2]. Unfortunately, pancreatic cancer is usually not detected until the late stages, with metastatic pancreatic cancer counting for about 52% of patients [14]. In contrast, while early-stage resectable pancreatic cancer has a much better five-year survival rate of 39%, only 11% of patients are detected at this stage [15]. Early detection of pancreatic cancer is highly valuable in improving pancreatic cancer survival; however, early detection is challenging, as there are no validated screening tests available for pancreatic cancer. Current efforts focus on risk stratification based on intraductal papillary mucinous neoplasms (IPMNs) and pancreatic intraepithelial neoplasia (PanIN) as well as familial risk factors [16]. Pancreatic lesions that are unlikely to progress to cancer may not be good candidates for surgical resection, as the operation is highly risky, while precancerous lesions may, as their prognosis is worse. Because of the potential toxicity and mortality associated with invasive biopsy of the pancreas, screening and early detection relies heavily on medical imaging. However, the small size of the early lesions/precursors and complex radiological appearances of these lesions and their background structures substantially challenge conventional radiology in providing reliable image-based early detection and diagnosis of precursor lesions. This offers a window of opportunity for novel quantitative imaging approaches. Both radiomics and deep learning methods have been applied in these types of applications.

As listed in Table 1, a series of papers were identified applying radiomics in pancreatic precancerous lesion diagnosis. As discussed in the introduction, manual segmentation was used in most of these studies. In the table, we list the number of observers, and for automatic and semi-automatic segmentation the software or algorithm used to automate the segmentation. Readers are referred to the original publications for additional details on segmentation. Standard-of-care imaging modalities (primarily CT) were used in these studies, though other modalities such as PET were used as well. For example, several radiomic models were developed to diagnose and evaluate the malignancy of cysts, showing improved accuracy compared to conventional radiology [17,18,19,20,21,22,23,24,25,26,27,28,29,30]. Pancreatic cysts have a fairly common occurrence relative to pancreatic cancer in adults; prevalence increases with age, and can show up as incidental image findings or on screening images. Because these cystic lesions are correlated with a wide range of histologic differentiation and malignant risk, image diagnosis distinguishing among them represents a crucial challenge. The conventional radiological diagnosis of pancreatic cysts is only accurate 60–70% of the time [31]. Using quantitative approaches, studies have been able to create a radiomics-based model in order to differentiate cyst types and propose risk stratification useful in determining treatment. High-risk lesions are recommended as candidates for surgical resection, while low-risk lesions are recommended as candidates for less aggressive management. For example, Wei et al. retrospectively studied CT-based radiomics on 260 patients with pancreatic cystic neoplasm and who underwent a pancreatic resection for it [20]. Using the pathology-established diagnosis of these 260 lesions, the authors grouped them into benign serous cystic neoplasms (SCNs) and malignant non-SCNs, the latter consisting of IMPNs, mucinous cystic neoplasms, and solid pseudopapillary neoplasms. With cross-validation in the training cohort of 200 patients, their radiomic model achieved an area under the receiver operating characteristic curve (AUC) of 0.84 on the independent validation of 60 patients. In contrast, only 30% (31 of 102) of the SCNs were correctly diagnosed pre-surgery with conventional radiology; thus, radiomics clearly shows potential as a computer-aided diagnosis (CAD) tool for improving the efficiency and accuracy of pancreatic cyst diagnosis. In another study using 53 patients with surgically resected IPMNs, Hanania et al. showed that a radiomic model based on texture features could differentiate low-grade versus high-grade IPMNs for the risk stratification necessary in clinical workflows and treatment decisions, with a high AUC of 0.96 in cross-validation compared with a false positive rate of 36% using the clinical Fukuoka criteria [17]. Huang et al. were able to use a radiomic model to predict invasive behavior in pancreatic solid pseudopapillary neoplasms [32]. Song et al. used a radiomic model to predict the recurrence risk of pancreatic neuroendocrine neoplasms [33]. Watson et al. applied a deep learning model to predict the malignancy of pancreatic cystic neoplasms [34]. Surgery is the only treatment for these neoplasms, and using radiomic and deep learning models can help to predict the prognosis and therefore led to a more accurate clinical decision regarding surgical resection. The current progress on texture analysis of pancreatic lesions for differential diagnosis has been reported in a recent review by Awe et al. [35].

Deep learning models are able to differentiate and risk stratify precancerous pancreatic lesions as well, as summarized in Table 2. Similar to radiomics studies, most of these studies were based on CT, though other modalities such as MRI and EUS were studied as well. Several studies adopted CNNs to predict lesion or cyst diagnosis and malignancy, such as lesion type or grade [37,38,39,40,41,42,43]. For example, using the EUS images of 206 patients with IPMNs that were later surgically resected, Kuwahara et al. developed a CNN model that achieved 94% accuracy for IPMN malignancy diagnosis, compared with 56% accuracy of human diagnosis [43]. In another study, Corral et al. used CNN to classify IPMN based on MRI images, and achieved a comparable AUC of 0.78, compared with 0.76 using the American Gastroenterology Association guidelines and 0.77 using the Fukuoka criteria [42]. In addition to these studies that used deep learning models alone, other studies combined deep learning with radiomics by adding radiomic features to the input channels of the deep learning algorithm, and others created fusion models (ensemble models) to integrate radiomics-based and deep learning-based predictions. Dmitriev et al. presented such a study [41]; using CT images of 134 patients with pancreatic cysts consisting of four histopathological types, they trained a radiomics model, a CNN model, and an ensemble/fusion model to classify the cyst lesion types [41]. On cross-validation, the radiomics model and the CNN model achieved an overall accuracy of 79.8% and 77.6%, respectively, while the ensemble/fusion model reached 83.6% [41]. Fusion models outperform radiomic models and deep learning models in these early lesion classification and malignancy diagnosis applications.

### 3.2. Pancreatic Cancer Detection and Diagnosis

Because reliable pancreatic cancer detection and diagnosis is unattainable based simply on symptoms and signs, medical imaging plays an essential role. A variety of imaging modalities can be used, including transabdominal US, CT, ERCP, MRCP, etc. CT is the most commonly used imaging modality among these, with a reported sensitivity of detection ranging from mid~70% to high~90% [47]. However, the accuracy of detection and diagnosis is highly dependent on the radiologist’s experience; misdiagnosis and missed diagnosis are not uncommon. Therefore, radiomics and deep learning have been explored to aid the clinical task of image-based pancreatic cancer detection and diagnosis. Table 3 and Table 4 summarize radiomics and deep learning research on these applications, respectively.

For detection, several works have shown the utility of radiomic models in differentiating pancreatic cancer tissue and healthy tissue [54,55]. Chu et al. trained a whole-pancreas ROI-based radiomic model on 225 training cases and validated the model on 125 validation cases [55]; the resulting model consisted of 40 radiomic features and achieved a very high AUC of 99.9% [55]. Chen et al. applied radiomic features and machine learning to investigate the utility of radiomics modeling in detecting pancreatic cancer [54]. Based on contrast-enhanced CT, they observed that pancreatic cancer tends to be hypodense and more heterogeneous compared with normal pancreas, as reflected by the relevant radiomic feature values. Quantitatively, their radiomics model trained on >1000 subjects achieved AUCs of 0.98 and 0.91 on local and external test datasets.

Deep learning models have proven useful for detecting pancreatic cancer. Zhang et al. used a novel deep learning framework consisting of Augmented Feature Pyramid networks, Self-adaptive Feature Fusion, and a Dependencies Computation Module to detect pancreatic cancer tumors, which resulted in an AUC of 0.95 on internal testing [77]. In a large-cohort study, Liu et al. applied CNN-based modeling on contrast-enhanced CTs of ~700 subjects (~1:1 with pancreatic cancer vs. healthy pancreas controls, divided 4:1 into training and validation sets) [70]. The model was further tested on a ~200-subject independent local cohort and a ~350-subject independent international cohort, achieving an AUC of 1.00 and 0.92, respectively [70]. For their local validation and testing datasets, the performance of the CNN model was compared against that of human radiologists and showed significantly higher sensitivity [70]. In another study, Chu et al. reported their initial experience of training deep learning networks to detect pancreatic adenocarcinoma [69]. They took a two-step approach to their curated large CT cohort of pancreatic cancer patients and controls with a healthy pancreas, using supervised learning to first train deep learning models to automatically segment all abdominal organs, and then to detect pancreatic cancer. Their algorithms achieved segmentation performances superior to published state-of-the-art algorithms, and showed 94.1% sensitivity and 98.5% specificity for pancreatic cancer detection in preliminary testing.

As pancreatic cancer is highly aggressive, correctly diagnosing it from benign or less-aggressive lesions could reduce unnecessary surgical resections that potentially lead to patient morbidity. Image-based grading and histopathology prediction can aid better treatment stratification. Reinert et al. identified CT-based textual features that can differentiate between pancreatic ductal adenocarcinoma and pancreatic neuroendocrine tumors as well as between low-grade and high-grade pancreatic neuroendocrine tumors [63]. Gu et al. were able to differentiate pancreatic ductal adenocarcinoma and neuroendocrine tumors from solid pseudopapillary neoplasm using MRI radiomic features [57]. Zhao et al. and Benedetti et al. used CT-based radiomic features to discriminate pancreatic neuroendocrine tumor grades, and Bendetti et al. predicted lymph node invasion status [48,68]. Similarly, using texture features on CT, Canellas et al. were able to differentiate grade 1 from grade 2 and 3 pancreatic neuroendocrine tumors [52]. In a large-cohort study, Chang et al. used contrast-enhanced CTs of ~300 patients to train and validate radiomics models to differentiate high-grade versus low-grade pancreatic ductal adenocarcinoma, achieving an AUC of 0.91 on the internal validation set and 0.77 on a 100-patient external testing cohort [53]. Other imaging modalities have been explored as well; in an early study, Zhu et al. used a support vector machine model on texture features from EUS images to differentiate pancreatic cancer from chronic pancreatitis [78]. With a total of ~400 patients, >90% accuracy was achieved in cross-validation. Based on various MRI sequences, Deng et al. tested radiomics models and compared them against a clinical model to differentiate pancreatic cancer from mass-forming chronic pancreatitis [56]. The radiomic models developed on a training cohort achieved performances much superior to the clinical model on a validation cohort from a different institution (AUCs of 0.88–0.96 vs. 0.65). Bevilacqua et al. used [68Ga]Ga-DOTANOC PET/CT radiomic features to detect grade 1 and 2 pancreatic neuroendocrine tumors [49].

Deep learning models have been successfully applied in pancreatic cancer diagnosis. CNN-based models have been frequently used. Si et al. applied ResNet models to diagnose different pancreatic lesions and achieved an average accuracy of 82.7% for all tumor types [74], and generated saliency maps to highlight the image regions relevant to the decision. Saftoiu et al. used an extended neural network analysis on EUS elastography for differential diagnosis of pancreatic cancer and chronic pancreatitis, and achieved an average testing performance of 95% on cross-validation [72]. Ziegelmayer et al. applied CNN modeling on CT images to discriminate between pancreatic cancer and autoimmune pancreatitis, and compared it with radiomics modeling [79]. On cross-validation, they achieved an average AUC of 0.90 with the deep learning model, outperforming their radiomic model, which had an AUC of 0.80.

Correctly detecting and diagnosing pancreatic cancer is crucial for managing the disease and selecting optimal treatment, and is ultimately crucial for patient outcomes. Accurate diagnosis of non-cancer versus cancer could reduce unnecessary surgical resections, which can lead to patient morbidity. Overall, both radiomic and deep learning models show great promise in these clinical tasks and could be implemented into computer-aided diagnosis systems for pancreatic cancer. These models could reduce the time and manual effort involved in these clinical tasks, reduce invasive biopsy procedures, and potentially offer more accurate diagnoses in order to improve treatment planning and improve patient outcomes.

### 3.3. Pancreatic Cancer Prognosis

Significant radiomic and deep learning features in pretreatment images have been used to predict treatment efficacy for pancreatic cancer. Survival can be predicted by both radiomic and deep learning models using pretreatment images in order to determine the level of treatment needed for the best chance of patient survival. Similarly, predictions on recurrence, metastasis, and surgical margins can be used to strategize regarding the optimal treatment for an individual patient.

Radiomic models have been used to predict progression-free survival, relapse-free survival, and overall survival for pancreatic cancer [80,81,82,83,84,85,86,87,88,89,90,91,92,93,94], and deep learning models have been used to model survival as well [95,96,97,98,99]. Mapelli et al. used a radiomic model to predict the aggressiveness of pancreatic neuroendocrine neoplasms [100]. Gao et al. used a deep learning CNN model to predict grades of PNET, with an average accuracy of 85.1% ± 0.4% and an average accuracy on the external validation set ranging between 79.1 and 82.4% [99]. Klimov et al. were able to predict metastasis risk in PNETs using a deep learning approach that had high ability to differentiate tumors, with an accuracy of >95% [101]. The models of both Gao et al. and Klimov et al. show promise in accurately assigning treatment plans to PNET patients while correctly predicting prognosis. Tang et al. were able to predict early recurrence in resectable pancreatic cancer using a radiomic nomogram [102]. Patients with a high risk of recurrence may be treated more aggressively or with other treatment modalities using the risk stratification proposed by the model developed by Tang et al. Bian et al. predicted the risk of lymph node metastasis in pancreatic cancer patients using a radiomic model [103]. Liu et al. were able to predict lymph node metastasis with their radiomic model as well [104]. If a pancreatic cancer patient already has lymph node metastases, a radical pancreatic operation may be futile and not worth the risks. The models developed by Bian et al. and Liu et al. could help to relieve patients from unnecessary operations.

In a different study, Bian et al. created a radiomic model to predict superior mesenteric vein resection margin (R1/2 vs. R0) in patients with pancreatic head cancer; their model was able to predict patient prognosis, as R1/2 resection is associated with poor overall survival relative to R0 resection [105]. Hui et al. were able to predict resection margin for pancreatic head cancer using a radiomic model as well [106]. Zhang et al. created a radiomics model that could predict postoperative pancreatic fistula in patients undergoing pancreaticoduodenectomy; their model could help with decisionmaking regarding risks of a pancreaticoduodenectomy [107]. Postoperative pancreatic fistula is one of the more harmful consequences of a pancreatic resection or pancreaticoduodenectomy, and the use of any of the aforementioned models could help to predict patient prognosis and assist with clinical decision making.

Li et al. created a radiomic model that could predict CD8+ tumor-infiltrating lymphocyte expression levels in patients with pancreatic ductal adenocarcinoma [107]. Patients with high levels of CD8+ tumor-infiltrating lymphocyte expression are possibly able to undergo immunotherapy targeting immune checkpoint inhibitors to improve their prognosis [108]. Bian et al. were able to predict tumor-infiltrating lymphocyte expression in their radiomic and deep learning models as well [109]. These kinds of models, which can predict patient prognosis, lead to better treatment predictions, and ultimately improve the clinical decisionmaking process, are summarized in Table 5 and Table 6.

### 3.4. Treatment Stratification, Delta-Radiomics, and Radiogenomics

Apart from diagnosis and differentiation of pancreatic diseases, radiomics can play a helpful role in predicting optimal therapy paths. Several studies in radiomics have predicted treatment response to pancreatic cancer [81,111,114,115,116,117,118,119,120]. Parr et al. applied radiomic models to pretreatment CT images in order to predict overall survival and locoregional recurrence of pancreatic cancer after stereotactic body radiation therapy [81]. Their radiomic model and the model combining radiomic and clinical features outperformed the pure clinical model in these predictions, with an average concordance index of 0.66 and 0.68 versus 0.54 for survival and an average AUC of 0.78 and 0.77 vs. 0.66 for recurrence on validations [81]. Cozzi et al., using a hybrid clinical–radiomics model, were able to differentiate high and low risk in terms of overall survival for patients treated with stereotactic body radiation therapy, with an AUC of 0.81 [114]. Those patients with a low overall survival prediction may need more aggressive treatment, and with this model high-risk and low-risk groups can be more accurately identified. Watson et al. applied deep learning CNN to predict pathologic tumor response to neoadjuvant therapy in pancreatic cancer, with an AUC of 0.738 in predicting response to chemotherapy and an accuracy of 78.3% in predicting response to resectability [121].

Another direction of radiomics, as in studies on other cancers, is delta-radiomics. Delta-radiomics assesses the temporal change or kinetics of radiomic signatures and explores its value in evaluating tumor progression or predicting long-term patient outcomes. The examined temporal window using delta-radiomics can be relatively short, such as when using daily imaging during a radiation therapy treatment course, or more extended, as when using periodical imaging from diagnosis to treatment and follow-up. Delta-radiomics has been explored for pancreatic cancer thanks to its value in prognosis prediction and treatment stratification. Using daily imaging during a radiation therapy treatment course, Chen et al. showed that patients with good pathological pancreatic tumor response tended to have large changes in certain radiomic features of the tumor compared to those with poor tumor response; radiation-induced delta-radiomics may potentially be used for early assessment of treatment response during radiation delivery [122].

Aside from the above clinical applications, radiomics has been used in radiogenomics. Radiogenomics is an offshoot branch of radiomics that applies radiomic workflow and imaging features coupled with genomic profiles [123] to assess the association between imaging phenotypes and the underlying tumor biology. These radiomic signatures associated with underlying patterns of gene expression can then be used to predict prognosis and optimal treatment. While radiogenomics has been more widely studied for other types of cancer, pancreatic cancer radiogenomic studies remain sparse. On the other hand, the genomic landscape of pancreatic cancer is diverse and many mutations have been detected, creating many potential opportunities for radiogenomic analysis [36]. A few radiogenomic studies have been conducted [8,9,36,124,125]. Katabathina et al. suggest that the varied biological tumor features related to the different mutations in panNENs may result in morphological changes that are appreciable with imaging [126]. McGovern et al. identified CT-based radiomic features that are significantly associated with the alternative lengthening of the telomere phenotype of pancreatic neuroendocrine tumors [36]. Attiyeh et al. demonstrated that radiomic signatures of preoperative CT can predict the mutation status of certain pancreatic cancer driver genes, such as SMAD4 [36]. The use of radiogenomics can increase personalized medicine within pancreatic cancer patients, leading to better outcomes. Table 7 summarizes the applications of both radiomics and deep learning in treatment stratification, delta-radiomics, and radiogenomics, respectively.

## 4. Limitations and Future Directions

While quantitative imaging approaches with radiomics and deep learning show promise in assisting and improving a range of clinical applications for combating pancreatic cancer, they do have several limitations.

One limitation is the inherent uncertainty and heterogeneity in image acquisition. As these quantitative imaging methods aim at identifying tumor- or lesion-specific characteristics among different patients, variations in image acquisition among patients, such as differences in makes and models of scanners or in acquisition protocols and reconstruction parameters, can act as confounding factors. The acquisition hardware, software, procedures, operators, and measurement methods may all contribute to these variabilities. In addition to image acquisition uncertainty common to other diseases, for pancreatic cancer respiration-related motion uncertainty needs to be considered, as pancreatic tumors can undergo considerable motion correlated with breathing. Using 4D or breath-hold CTs, or excluding features sensitive to these motions, may be necessary for the workflow [126]. Thus far, studies have been conducted to assess these sources of uncertainties and their impacts and to develop mitigation strategies, although more such studies are needed [91,127]. The best way to mitigate image acquisition-related uncertainty is to design prospective trials that use uniform acquisition and reconstruction parameters with a single scanner. This needs to be a conscious effort on the part of the entire clinical and research community, and imaging acquisition should be an integral part of standardization for future clinical trials. For retrospective datasets with heterogeneous image acquisition, mitigation strategies include applying voxel, gray-level, and bit-depth resampling, applying smoothing filters, and using test–retest datasets and other uncertainty study findings to exclude features sensitive or unstable to these variabilities.

Image processing is a necessary step in the quantitative imaging workflow. Lack or improper execution of processing steps can lead to the degradation of the subsequent radiomic or deep learning models under development. Image resampling and normalization are important processing steps, and should be optimized for the clinical and modeling task at hand as well as for the images being used. For example, CT image intensities tend to be more stable across different scanners and scanning protocols due to the common Hounsfield Unit definition, whereas MR image intensities are much more variable even for the same type of image sequences. Applying field bias correction and appropriate normalization is therefore essential for MR-based studies. Currently, there is a need for more investigations to assess the impact of and optimize image processing for both radiomics and deep learning applications.

For radiomics modeling, ROI segmentation as an upper-stream step of the workflow adds additional uncertainty due to inter-observer and intra-observer variations. For segmentation variabilities, having multiple observers and repeated segmentations may help reduce the segmentation-related uncertainty, as does employing automatic or semi-automatic segmentation. It is important to note that although the above-mentioned uncertainty sources exist for all radiomics and deep learning applications, their effects may vary for different diseases and clinical applications. For pancreatic cancer, segmentation uncertainty may play a greater role in the overall uncertainty than for diseases such as lung cancer where the tumor can be far more easily and accurately delineated. In addition to assessing the effects of inter-observer segmentation variation on pancreatic cancer radiomics, Wong et al. identified an interdisciplinary variation between radiation oncology and radiology, possibly attributable to discipline-specific training differences [128]. On the other hand, Yamashita et al. found in their study that variations in CT scans affected radiomics reproducibility to a greater extent than segmentation variation [129]. Automatic or semi-automatic segmentation helps to improve both the consistency and efficiency of ROI segmentation. However, developing effective and accurate automatic segmentation methods is particularly challenging for pancreatic cancer as the lesion and its abdominal background can both appear heterogeneous and similar in intensity on images. Therefore, algorithms that have found good success for cancers such as lung and liver cancer are unable to achieve similar segmentation accuracy for pancreatic cancer. Thus far, the utilization of automatic or semi-automatic segmentation has been largely limited to PET-based quantitative imaging studies, as in that situation the volumes can be more easily defined via thresholding based on uptake values rather than on anatomy. In more recent years, there has been progress in both normal and cancerous pancreatic auto-segmentation algorithms, especially with the help of deep learning [130].

For deep learning studies, one limitation is a lack of interpretability and transparency. Avanzo et al. suggests that machine learning and deep learning are less intuitive than radiomics models alone [10]. Manually-crafted radiomic features are attributable to physical properties, and partially resemble the qualitative and descriptive image features conventionally used by human experts. On the other hand, deep features are often something of a black box. One continuing effort of deep learning studies is to improve the explainability and transparency of the models. Strategies such as dimension reduction, feature importance, attention mechanisms, knowledge distillation, and surrogate models have been developed, although further work is needed [131]. New fusion models incorporating the intuitiveness of radiomic features with the accuracy of deep learning models are becoming increasingly popular, and show promise in predictive accuracy; however, their use remains limited. Overall, more studies focusing on pancreatic diseases with these quantitative imaging approaches are warranted in order to provide further evidence supporting their clinical promise.

Aside from these general limitations and challenges of quantitative imaging methods, pancreatic cancer presents several major challenges of its own, chiefly the limited dataset size for such studies. The reason is twofold: first, the prognosis of pancreatic cancer is very poor and patients deteriorate rapidly; second, pancreatic cancer has a relatively low incidence compared with other major cancers. Therefore, there are usually limited available patient populations at a given institution, and single-institutional studies, which make up most of the existing research, are often conducted on relatively small groups of patients compared to other cancers. Due to these limitations, the accuracy and predictive power of radiomics and deep learning for pancreatic cancer are relatively low.

Another limitation is the lack of standardization in study design and analysis workflow across different studies, often making the quantitative findings from individual studies difficult to compare against one another. Most existing studies are retrospective studies and many have small patient populations. For these studies with smaller sample sizes, model overfitting tends to be an issue if the study is not carefully and rigorously designed, leading to the inflating of model performance in studies which can be difficult to reproduce or generalize. Due to variation in the rigor and design of studies, a higher AUC reported in one study may not represent a superior model to one with a lower AUC reported for the same clinical application by another study. To this end, Gillies et al. suggest that establishing benchmarks for the conduct of radiomic studies could help to solve the challenge of reproducibility [132]. In addition, the Radiomics Quality Score (RQS), established in 2017 by Lambin et al., could help with the uncertainties associated with performing radiomic studies [133]. The RQS is a 36-point scale that either rewards or penalizes studies based on their methodology, analysis, and reporting with the ultimate purpose of encouraging the best scientific practices and helping to standardize the radiomic workflow [133]. The use of the RQS could help to promote standardization within the radiomic field and make the quantitative results from different studies more comparable.

As many fields of cancer research have adopted the use of computer-aided diagnosis and prediction models, these are desperately needed within the clinical realm of pancreatic cancer in order to push back against this vicious disease. While these studies show promise for “phenotyping” tumors and pancreatic disease prognosis, continued research is needed to resolve the issue of unresectable pancreatic cancer prognosis [134]. While radiomics and deep learning models have shown promise for the diagnosis, prognosis, and prediction of optimal treatment of pancreatic diseases, there is a need for further and better studies. Both radiomics and deep learning approaches rely on large sample sizes and high-quality data. Prospective studies with standardized and optimized image acquisition and processing remain highly desirable. There is a need for normalization within the radiomics and deep learning workflow as well, in order to reduce uncertainties, increase reproducibility, and benchmark results across different studies. Requiring studies to make their data and algorithms publicly available can help to increase the resulting transparency of a study and increase data availability for future studies. As these methods demonstrate the great potential of imaging data science in combating pancreatic cancer, continuing efforts need to be made to increase the interpretability and generalizability of the models and to promote clinical confidence and trust.

## 5. Conclusions

In this review, we have discussed the use of radiomics and deep learning as regards pancreatic cancer. Starting with a clinical introduction to pancreatic cancer and technical overviews of these two quantitative imaging analysis methods, clinical applications were reviewed for precancerous lesion diagnosis, pancreatic cancer detection and diagnosis, prognosis prediction, treatment stratification, and radiogenomics. The limitations of the current models and approaches were discussed along with possible solutions and potential future directions. Despite the discussed limitations, there is clear evidence of the promise of radiomics and deep learning models in the clinical workplace. This review provides a comprehensive literature summary and discussion for both researchers and clinicians interested in radiomics and deep learning applications in pancreatic cancer.

## Figures and Tables

**Table 1:** Radiomics studies in diagnosing precancerous pancreatic diseases.

Reference	Image	Software	Endpoints	Segmentation Process (Number of Readers)	Sample Size (Training + Validation)	Number of Features Extracted	Results
Attiyeh et al. [36]	CT	In-house software in MATLAB	BD-IPMN risk	manual (1)	103 (10-fold cross-validation)	255	AUC = 0.79 for radiomics + clinical model vs. AUC = 0.67 for clinical model.
Chakraborty et al. [22]	CT	In-house software in MATLAB	BD-IPMN risk	manual (1)	103 (10-fold cross-validation)	150	AUC = 0.77 for radiomics model and AUC = 0.81 for combined radiomics and clinical model.
Cheng et al. [29]	CT and MRI	ITK-SNAP software and Artificial Intelligence Kit software	predicting the malignant potential of intraductal papillary mucinous neoplasms (IPMNs)	manual (2)	60	1037	MRI radiomics models achieved improved AUCs (0.879 with LR and 0.940 with SVM, respectively), than that of CT radiomics models (0.811 with LR and 0.864 with SVM, respectively). All radiomics models provided better predictive performance than the clinical and imaging model (AUC = 0.764).
Cui et al. [26]	MRI	MITK software	Low vs. high-grade in BD-IPMNs	manual (2)	103 + 48/51 (validation1/validation2)	328	Radiomics model: AUC = 0.836 (training); AUC = 0.811 (validation1); AUC = 0.822 (validation 2). Radiomics + clinical model: AUC = 0.903 (training); AUC = 0.884 (validation1); AUC = 0.876 (validation 2).
D‘Onofrio et al. [30]	MRI	MevisLasb and MATLAB	Identification and classification of IPMNs	manual (1)	91	<20	Entropy of the ADC map was found to correlate with tumor dysplasia (*p* = 0.034, AUC = 0.729)
Hanania et al. [17]	CT	IBEX	High-grade vs. low-grade IPMNs	Manual (2)	53	360	Best univariate AUC = 0.82
Harrington et al. [23]	CT	In-house software in MATLAB	IPMN risk	manual (1)	33	<20	AUC = 0.74 (cyst fluid inflammatory markers model) vs. AUC = 0.83 (radiomics model) vs. AUC = 0.91 (tumor-associated neutrophils model)
Huang et al. (2021) [32]	CT	Pyradiomics	Invasiveness of SPN	Manual (2)	85	1316	Best AUC = 0.914 on 3D-arterial model (compared vs. 2D and venous)
Polk et al. [28]	CT	Healthmyne	Malignancy of IPMNs	semi-automatic (1, Healthmyne software)	51 (5-fold cross-validation)	39	AUC = 0.87 (arterial model) vs. AUC = 0.83 (venous model) vs. AUC = 0.90 (combined)
Tobaly et al. [18]	CT	Pyradiomics	Differentiating IPMN grades	Manual (1)	296 + 112	107	AUC = 0.84 in training set and AUC = 0.71 in validation
Wei et al. [20]	CT	unknown	Computer-aided diagnosis of SCN	Manual (2)	200 + 60	385	AUC = 0.767 in training and AUC = 0.837 in validation
Xie et al. [21]	CT	In-house algorithm in MATLAB	Differentiating MCN vs. MaSCA	Manual (1)	57	1942	AUC = 0.989 (radiomics model) vs. AUC = 0.775 (radiological model) vs. AUC = 0.994 (combined model) on bootstrapping
Xie et al. [27]	CT	Pyradiomics	MCN vs. ASCN	semi-manually (1, 3D Slicer)	216 (10-fold cross-validation)	764	Average AUC = 0.784 (radiomics model) vs. AUC = 0.734 (clinical model)
Yang et al. [37]	CT	LifeX	Differentiating SCA vs. MCA	manual (2)	78 (4:1)	unknown	Slice thickness = 2 mm: AUC = 0.77 in training and AUC = 0.66 in validation; Slice thickness = 5 mm: AUC = 0.72 in training and AUC = 0.75 in validation

Abbreviations used in this table: Atypical Serous Cystadenomas (ASCN), Branch-Ductal Intraductal Papillary Mucinous Neoplasm (BD-IPMN), Intraductal Papillary Mucinous Neoplasm (IPMN), Macrocystic Serous Cystadenoma (MaSCA), Mucinous Cystadenomas (MCA), Mucinous Cystic Neoplasm (MCN), Neuroendocrine Tumor (NET), Pancreatic Neuroendocrine Neoplasm (PanNEN), Serous Cystadenomas (SCA), Serous Cystic Neoplasms (SCN), Solid Pseudopapillary Neoplasm (SPN).

**Table 2:** Deep learning studies in diagnosing precancerous pancreatic diseases.

Reference	Image	Software	Endpoints	Sample Size (Training + Validation)	Results
Abel et al. [44]	CT	Two-step nnU-Net architecture	Detection of PCL	221 (5-fold cross validation)	Mean sensitivity = 78.8% (87.8% for cysts ≥220 mm^3^ and 96.2% for lesions in distal pancreas)
Dmitriev et al. [41]	CT	CNN	Classification of 4 types of cysts: IPMN, MCN, SCA, SPN	134 (10-fold cross validation)	Accuracy = 83.6% for the ensemble classifier (RF + CNN)
Luo et al. [40]	CT	CNN (ResNet50)	PNEN grading	93 (8-fold cross validation) + 19 (independent testing set)	AUC = 0.81 (validation) AUC = 0.82 (testing)
Nguon et al. [45]	EUS	CNN using ResNet50	MCNs vs. SCNs	89 + 20 (holdout validation)	AUC = 0.88 for the classification of pancreatic SCNs and MCNs
Watson et al. [34]	CT	CNN (LeNet architecture)	PCN malignancy	18 + 9	AUC = 0.966 in high-risk lesions
Yang et al. [46]	CT	MMRF-ResNet	MCNs vs. SCNs	110 (80:20 total images)	AUC = 0.96 for the classification of pancreatic SCNs and MCNs
Song et al. [33]	CT	* Fusion model. In-house software (manual segmentation by two observers, 143 radiomic features)	panNEN post-surgical recurrence risk	56 + 18	Better validation performance on arterial models with AUC = 0.77 (radiomics/DL fusion models) and AUC = 0.56 (radiomics model), compared to venous.

Abbreviations used in this table: Convolutional Neural Network (CNN), Intraductal Papillary Mucinous Neoplasm (IPMN), Mucinous Cystic Neoplasm (MCN), Multi-channel-Multiclassifier-Random Forest (MMRF), Pancreatic Cystic Lesion (PCL), Pancreatic Cystic Neoplasm (PCN), Pancreatic Neuroendocrine Neoplasm (PanNEN), Random Forest (RF), Serous Cystadenomas (SCA), Serous Cystic Neoplasm (SC.N).* Combined both a radiomic analysis and a machine learning analysis.

**Table 3:** Radiomics studies in pancreatic cancer detection and diagnosis.

Reference	Image	Software	Endpoints	Segmentation Process (Number of Readers)	Sample Size (Training + Validation)	Number of Features Extracted	Results
Benedetti et al. [48]	CT	In house with Matlab	Discriminating histopathologic characteristics of PNET	Manual (1)	39	69	Best AUC = 0.86
Bevilacqua et al. [49]	PET/CT	In house with Matlab	Grade 1 vs. 2 primary PNET	Manual (1)	25 + 26 (model A) 26 + 25 (model B) 51 (model C)	60	Best performance was achieved by model A test AUC = 0.90
Bian et al. [50]	MRI	Pyradiomics	G1 vs. G2/3 grades in patients with PNETs	Manual (2)	157	1409	AUC = 0.775
Bian et al. [51]	MRI	Pyradiomics	PNET grades	Manual (1)	97 + 42	3328	AUC = 0.851 (training) AUC = 0.736 (validation)
Canellas et al. [52]	CT	TexRAD	Differentiating PNET grades	Manual (2)	101	36	Accuracy of 79.3% for differentiating grade1 vs. grades 2/3.
Chang et al. [53]	CT	IBEX	Histological grades of PDAC	Manual (2)	151 + 150 (local) +100 (external validation)	1452	AUCs = 0.961 (training), AUC = 0.910 (local validation), and AUC = 0.770 (external validation)
Chen et al. [54]	CT	Pyradiomics	Differentiating PDAC from normal pancreas	Manual (2)	915 + 200 (local test) + 264 (external test)	88	AUC = 0.98 (local test) AUC = 0.91 (external test)
Chu et al. [55]	CT	Pyradiomics	Differentiating PDAC from normal pancreas	Manual (3)	255 + 125	478	AUC = 0.999
Deng et al. [56]	MRI	IBEX	DifferentiatingPDAC and MFCP lesions	Manual (2)	64 + 55	410	AUCs for the T1WI, T2WI, A and, P and clinical models were 0.893, 0.911, 0.958, 0.997 and 0.516 in the primary cohort, and 0.882, 0.902, 0.920, 0.962 and 0.649 in the validation cohort.
Gu et al. [57]	MRI	Artificial Intelligence Kit	SPN vs. differential diseases (PDAC, NET, and cystadenoma)	manual (2)	48 + 113	2376	In validation, AUC = 0.853 for T2 (best performing single sequence), AUC = 0.925 for multi-parametric MRI radiomics model, and AUC = 0.962 for radiomics + clinical model.
Li et al. [58]	CT	Fire Voxel	Atypical PNET vs. PDAC	Manual (2)	75	<20	Best AUC = 0.887
Linning et al. [59]	CT	In house with Matlab	PDAC vs. autoimmune pancreatitis	Manual (2)	96 (5-fold cross validation)	1160	AUC = 0.977
Liu et al. [60]	PET/CT	Pyradiomics	PDAC vs. autoimmune pancreatitis	Manual (2)	112 (10-fold cross validation)	502	AUC= 0.967
Liu et al. [61]	CT and MRI	Pyradiomics	PNET grades	Manual (2)	82 + 41	1209	AUC = 0.92 (training) AUC = 0.85 (validation)
Park et al. [62]	CT	Pyradiomics	PDAC vs. autoimmune pancreatitis	Manual (4)	120 + 62	431	AUC = 0.975
Reinert et al. [63]	CT	Pyradiomics	Differentiating PDAC from PanNEN	Manual (1)	95	92	8 features highly significant (*p* < 0.005)
Ren et al. [64]	CT	Analysis Kit software	Pancreatic adenosquamous carcinoma vs. PDAC	Manual (1)	112 7:3 ratio	792	Average AUC of 0.82
Song et al. [65]	MRI	Pyradiomics	Differentiating NF-PNET and SPN	Manual (2)	79 (7:3 ratio)	396	AUC = 0.978 (radiomics) and AUC = 0.965 (radiomics + clinical) in the training set AUC = 0.907 (radiomics) and AUC = 0.920 (radiomics + clinical) in the validation set
Xing et al. [66]	PET/CT	Pyradiomics	Pathological grades in PDAC	Manual (2)	99 + 50	about 3000	AUC o = 0.994 (training) AUC = 0.921 (validation)
Zhang et al. [67]	CT	LifeX	Pathological grades of PNETs	Manual (3)	82 3:1 ratio	40	AUC = 0.82 (G1 vs. G2), 0.70 (G2 vs. G3), and 0.85 (G1 vs. G3), respectively
Zhao et al. [68]	CT	In house with Matlab	Grade 1 vs. 2 in PNET	Manual (2)	59 + 40	585	AUC = 0.968 (training) AUC= 0.876 (validation)

Abbreviations used in this table: Area Under Curve (AUC), Mass-forming Chronic Pancreatitis (MFCP), Pancreatic Neuroendocrine Neoplasm (PanNEN), Pancreatic Adenocarcinoma (PDAC), Neuroendocrine Tumor (NET) or Pancreatic Neuroendocrine Tumor (PNET), Solid Pseudopapillary Neoplasm (SPN).

**Table 4:** Deep learning studies in pancreatic cancer detection and diagnosis.

Reference	Image	Software	Endpoints	Sample Size (Training + Validation)	Results
Chu et al. [69]	CT	Deeply supervised nets with encoder-decoder architecture	PDAC detection	456	Sensitivity = 94.1%, specificity = 98.5%
Liu et al. [70]	CT	CNN	Differentiating pancreatic cancer vs. normal pancreas	295 + 691 (local test 1 + local test 2 + external test)	AUC = 0.997 (local test 1) AUC = 0.999 (local test 2) AUC = 0.920 (external test)
Ozkan et al. [71]	EUS	ANN with Relief-F feature reduction method	Pancreatic cancer diagnosis for different age groups	260 + 72	Age groups in years: <40, 40–60, >60: accuracy = 92%, 88.5%, 91.7%, respectively all age groups: accuracy = 87.5%
Săftoiu et al. [72]	EUS	ANN (MLP)	Differential diagnosis of chronic pancreatitis and pancreatic cancer	68 (10-fold cross validation)	Benign vs. malignant pancreatic lesions: AUC = 0.957 Chronic pancreatitis vs. pancreatic cancer: AUC = 0.965
Săftoiu et al. [73]	EUS	ANN (MLP)	Diagnosis of focal pancreatic masses	258 (10-fold cross validation)	Average AUC = 0.94 over 100 runs of a complete cross-validation cycle
Si et al. [74]	CT	CNN ResNet18 (pancreas location), U-Net32 (pancreas segmentation), ResNet34 (pancreatic tumor diagnosis)	Fully automated diagnosis of pancreatic tumors	319 + 347	AUC = 0.871 on testing for detection of all tumor types
Tonozuka et al. [75]	EUS	CNN	PDAC detection	92 (10-fold cross validation) + 47	AUC = 0.924 (cross validation) AUC = 940 (test)
Zhang et al. [76]	CT	Faster R-CNN combined with Feature Pyramid Network for feature extraction	Pancreatic tumor detection	2650 + 240 (images)	AUC = 0.946

Abbreviations used in this table: Artificial Neural Network (ANN), Area Under Curve (AUC), Convolutional Neural Network (CNN), Multilayer Perceptron (MLP), Pancreatic Adenocarcinoma (PDAC).

**Table 5:** Radiomics studies in pancreatic cancer prognosis.

Reference	Image	Software	Endpoints	Segmentation Process (Number of Readers)	Sample Size (Training + Validation)	Number of Features Extracted	Results
Bian et al. [103]	CT	Pyradiomics	Lymph node metastasis in PDAC	Manual (2)	225 (10-fold cross validation)	1029	Multivariate *p* < 0.0001
Bian et al. [105]	CT	Pyradiomics	R0 vs. R1 margin in pancreatic head cancer	Manual (2)	181 (10-fold cross validation)	1029	AUC = 0.750
Bian et al. [109]	MRI	Pyradiomics	Tumor-infiltrating lymphocytes in patients with PDAC	Manual (2)	116 + 40	1409	training AUC = 0.86 and validation sets AUC = 0.79
Cassinottoet al. [110]	CT	TexRAD	Disease-free survival in patients with resectable PDAC	Manual (1)	99	<20 (texture)	AUC 0.71
Cen et al. [84]	CT	Analysis Kit software	Stage I-II vs. III-IV PDAC and predict overall survival	Manual (2)	94 + 41	384	Training cohort AUC = 0.940 Validation cohort AUC = 0.912
Cheng et al. [80]	CT	TexRAD	Progression-free survival and overall survival in patients with unresectable PDAC	Manual (1)	41	<20 (texture)	AUC = 0.756
Cusumano et al. [83]	MRI	MODDICOM software	One-year local control in patients with locally advanced pancreatic cancer	Manual (2)	35 (5-fold cross validation)	368 radiomic features and 276 delta features	AUC = 0.78
D’Onofrio et al. [93]	CT	In house with unknown software	Metastatic vs. non-metastatic PDAC	Manual (1)	288	<20	Significant univariate features identified: size, arterial index, perfusion index, and permeability index (*p* < 0.05).
Eilaghi et al. [111]	CT	In house with Matlab	Overall survival for PDAC after surgical resection	Semi-automatic (1, in-house ProCanVAS)	30	<20	Max AUC = 0.716 in univariate
Hang et al. [89]	CT	LifeX	Overall survival for pancreatic cancer with liver metastases	Manual (1)	39	36	Nomogram showed good discriminative ability (CI = 0.754).
Hui et al. [106]	CT	Rbio2.8	R0 or R1 margin in pancreatic head adenocarcinoma	Manual (2)	86 (leave-one-out cross validation)	23	AUC = 0.861
Kaissis et al. [95]	MRI	Pyradiomics	Survival and tumor subtype in PDAC	Manual (2)	102 (10-fold nested cross validation) + 30	1474	AUC = 0.93 in cross-validation AUC = 0.90 in independent validation
Khalvati et al. [86]	CT	Pyradiomics	Prognostic value of CT-derived radiomic features for resectable PDAC	Manual (2)	30 + 68	410	Validation cohort with *p*-value of 0.047
Kim et al. [85]	CT	In house with unknown software	predict prognosis after curative resection in pancreatic cancer	Manual (1)	116	<20 (GLRLM)	One feature with *p* = 0.025 for survival
Li et al. [82]	CT	Pyradiomics	Lymph node metastasis	Manual (2)	118 + 41	2041	Best AUC = 0.811
Li et al. [108]	CT	Pyradiomics	CD8+ tumor-infiltrating lymphocyte expression levels in patients with PDAC	Manual (2)	137 + 47	1409	Training set AUC = 0.75 and validation set AUC = 0.67
Liu et al. [104]	CT	Pyradiomics	Lymph node metastasis in resectable PDAC	Manual (2)	85	1124	AUC = 0.841 (radiomics) vs. AUC = 0.682 (conventional)
Mapelli et al. [100]	PET/CT	Chang-Gung Image Texture Analysis software package	PanNEN risks	Automatic with SUV thresholding (40% of SUVmax)	61	9	Four principal components extracted: PC1 correlated with all 18F-FDG variables, while PC2, PC3 and PC4 with 68Ga-DOTATOC variables. PC1 could predict angioinvasion (*p* = 0.0222); PC4 could predict lymph nodal involvement (*p* = 0.0151). All PCs except PC4 could predict tumor dimension
Mapelli et al. [94]	PET/CT	Chang-Gung Image Texture Analysis software package	PanNEN risks	Automatic with SUV thresholding (40% of SUVmax)	83	9	Individual parameters evaluated for various clinical risk endpoints
Mori et al. [90]	PET	Spaarc Pipeline for Automated Analysis and Radiomics Computing (SPAARC)	Distant-relapse-free-survival after radio-chemotherapy for locally advanced pancreatic cancer	Semi-automatic (gradient based, PET-Edge, MIM)	116 + 60	198	Training cohort *p* = 0.002 and validation cohort *p* = 0.03.
Salinas-Miranda et al. [91]	CT	Pyradiomics	Overall survival and time to progression; validate radiomic features developed in resectable PDAC on a test set of patients with unresectable PDAC undergoing chemotherapy	Manual (1)	0 + 108	2 previously developed features	One feature remained significant with a HR = 1.27 for overall survival and a HR of 1.25 for time to progression
Shi et al. [112]	CT	ITK-SNAP software and Artificial Intelligent Kit	Survival after upfront surgery in patients with PDAC	Manual (2)	210 + 89	792	CI = 0.74 in the training set and CI = 0.73 in the validation set.
Tang et al. [102]	MRI	AK software	Early recurrence in resectable pancreatic cancer	Manual (2)	123 + 54 (+126 external validation)	328	AUC = 0.871 (training cohort), AUC = 0.876 (internal validation cohort), and AUC = 0.846 (external validation cohort).
Toyama et al. [87]	PET	LifeX and machine learning algorithms	1-year survival	Semi-automatic (2, with SUV thresholding at 40% of SUVmax)	161 (10-fold cross validation on 138)	42	Best AUC = 0.720
Xie et al. [88]	CT	Mazda	Survival in patients with resected PDAC	Manual (3)	147 + 73	300	AUC = 0.701 in training cohort AUC = 0.715 in validation cohort
Zhang et al. [107]	CT	Pyradiomics	Postoperative pancreatic fistula after pancreaticoduodenectomy	Manual (2)	80 + 37	1219	AUC = 0.825 in training cohort and AUC = 0.761 in validation cohort

Abbreviations used in this table: Area Under the Receiver Operating Curve (AUC), Concordance Index (CI), Gray-level Run-length Matrix (GLRLM), and Pancreatic Adenocarcinoma (PDAC).

**Table 6:** Deep learning studies in pancreatic cancer prognosis.

Reference	Image	Software	Endpoints	Sample Size (Training + Validation)	Results
Gao et al. [99]	MRI	CNN combined with GAN for synthetic image generation	PNET grades	96 (5-fold cross validation) + 10	Micro-average AUC = 0.912 in internal validation set; Micro-average AUC = 0.845 in external validation set
Klimov et al. [101]	Whole-slide imaging of resected tissues	CNN for tissue annotation, 18 different ML models for metastasis prediction	Metastasis risk in PNET	89	Tissue annotation: per-tile accuracy > 95%, whole slide 79%; Metastasis prediction: hazard ratio 4.71
Li et al. [92]	CT	Fusion model (70 conventional features and 256 deep convolutional features) Matlab	Survival time in PDAC	111 (k-fold leave-one-out cross validation, k = 10, 20, 30, 40)	Average AUC = 0.90
Yao et al.(2020) [96]	CT	* Fusion model. Pyradiomics, CNN (CE- convLSTM, combined with 3D-ResNet18 as the encoder)	PDAC survival and surgical margin	205 (5-fold cross validation)	survival prediction: C-index = 0.705; resection margin prediction: balanced-accuracy = 0.736
Yao et al. [113]	CT	CNN	Survival of primary resectable PDAC	296 (4-fold nested cross validation)	1-year overall survival: AUC = 0.684; 2-year overall survival: AUC = 0.689
Zhang et al. [97]	CT	CNN-based transfer learning model	prognosis of overall survival in PDAC patients	68 (5-fold cross validation) + 30	AUC = 0.72 in training cohort; AUC = 0.81 in test cohort
Zhang et al. [98]	CT	* Fusion model. Pyradiomics. Random forest-based models trained from features extracted using traditional radiomics pipeline and transfer learning	Overall survival in PDAC	68 (10-fold cross validation) + 30	AUC = 0.84 in test cohort

Abbreviations used in this table: Area Under Curve (AUC), Convolutional Neural Network (CNN), Generative Adversarial Network (GAN), Machine Learning (ML), Pancreatic Adenocarcinoma (PDAC), Pancreatic Neuroendocrine Tumor (PNET).* Combined both a radiomic analysis and a machine learning analysis.

**Table 7:** Radiomics and deep learning studies in treatment stratification, delta-radiomics, and radiogenomics in pancreatic cancer.

Reference	Image	Software	Endpoints	Segmentation Process (Number of Readers)	Sample Size (Training + Validation)	Number of Features Extracted	Results
Borhani et al. [120]	CT	TexRAD	Histologic response to neoadjuvant CRT and disease-free survival in patients with potentially resectable PDAC	Manual (1)	39	<20 for each filter, 6 filters applied	Prognostic features identified for histological response (*p* < 0.05), biochemical response (*p* < 0.01) and disease-free survival (*p* = 0.001).
Chen et al. [122]	CT	In house with Matlab	Delta-radiomic change during CRT and pathology responses on 15 patients that undergone subsequent resections	Manual (1)	20	<20	*p* = 0.046, 0.058, 0.042, and 0.12 for MCTN, SD, skewness and kurtosis, respectively.
Cozzi et al. [114]	CT	LifeX	Overall survival after stereotactic body radiation therapy	Manual (1)	60 + 40	41	AUC = 0.81 for the training set and AUC = 0.73 for the validation set
Liang et al. [119]	MRI	Pyradiomics	Efficacy of S-1 (oral antitumor agent)	Semi-automatic (2, a generic automatic segmentation algorithm based on a 3D domain using a prototype software, Radiomics, Siemens)	31 + 15	110	T1WI_NGTDM_Strength and tumor location are independent predictors of the efficacy of S-1 in the training cohort (*p* = 0.005 and 0.013), but marginal in the validation cohort (*p* = 0.073 and 0.050).
Nasief et al. [116]	CT	IBEX	Delta-radiomic change and overall progression in patients undergone neoadjuvant CRT	Manual (1)	50 (leave-one-out cross validation) + 40 (external)	>1300	Best AUC = 0.94
Nasief et al. [117]	CT	IBEX	Delta-radiomic change and overall progression in patients undergone neoadjuvant CRT	Manual (1)	24	Over 1300	The Cox proportional multivariate hazard analysis showed that a treatment related decrease in CA19-9 levels (*p* = 0.031) and delta radiomics (*p* = 0.001) were predictors of survival.
Parr et al. [81]	CT	Pyradiomics	Overall survival and locoregional recurrence following stereotactic body radiation	Manual (2)	74 (3-fold cross validation)	841	Validation: Average CI of 0.66 (radiomics) vs. 0.54 (clinical) for survival; Average AUC of 0.78 (radiomics) vs. 0.66 (clinical) for recurrence.
Steinacker et al. [115]	CT	MintLesion	Overall progression in advanced pancreatic cancer treated with systemic therapy	Semi-automatic (1, mintLesion®.)	13	<20	Two significant univariate features identified: mean positivity of pixel values (*p* = 0.030 for progression); kurtosis (*p* = 0.008 for time to local tumor spread and *p* = 0.017 for systemic progression).
Watson et al. [121]	CT	CNN (based onLeNet architecture)	Pathologic tumor response to neoadjuvant therapy in pancreatic adenocarcinoma	NA (deep learning)	65 + 16	NA (deep learning)	AUC = 0.738 (DL), AUC = 0.564 (CA19-9), and AUC = 0.785 (combined)
Zhou et al. [118]	CT	In house with Matlab	Candidate selection for irradiation stent placement among patients with unresectable pancreatic cancer with malignant biliary obstruction	Manual (2)	74 + 32	620	CI = 0.791 (radiomics + clinical) vs. CI = 0.673 (clinical) in the training set; CI = 0.779 (radiomics + clinical) vs. CI = 0.667 (clinical) in the validation groups
Attiyeh et al. [36]	CT	Matlab	CT imaging phenotypes and genetic and biological characteristics PDAC	Manual (1)	35	255	Radiomics associated with SMAD4 status and the number of genes altered
Gao et al. [125]	MRI	Pyradiomics	TP53 mutation status	Manual (2)	57	558 2D and 994 3D features	AUC = 0.96
Iwatate et al. [9]	CT	Pyradiomics	Genetic information	Manual (2)	107	1037	Radiogenomics-predicted p53 mutations associated with poor prognosis (*p* = 0.02), whereas the predicted abnormal expression of PD-L1 was not significant (*p* = 0.10).
Lim et al. [8]	PET	Chang-Gung Image Texture Analysis	KRAS, SMAD4, TP53, and CDKN2A mutation status	Semi-automatic (3, gradient based, PET-Edge, MIM)	116 + 60	35	Features identified that associated with KRAS and SMAD4 gene mutations, but not with TP53 and CDKN2A gene mutations.
McGovern et al. [124]	CT	Unknown	Predicting the ALT phenotype in PNET patients	Manual (2)	121	<20	Univariate (*p* < 0.05) and multivariate features (*p* = 0.006) found.

## Abbreviations

Alternative Lengthening of Telomeres (ALT); Artificial Neural Network (ANN); Atypical Serous Cystadenomas (ASCN); Area Under Curve (AUC); Branch-Ductal Intraductal Papillary Mucinous Neoplasm (BD-IPMN); Computer-Aided Diagnosis (CAD); Concordance Index (CI); Convolutional Neural Network (CNN); Chemoradiation Therapy (CRT); Fluorine 18-fluorodeoxyglucose (FDG); Generative Adversarial Network (GAN); Gray-Level Co-occurrence Matrix (GLCM); Gray-Level Run-Length Matrix (GLRLM); Intraductal Papillary Mucinous Neoplasm (IPMN); Least Absolute Shrinkage and Selection Operator (LASSO); Macrocystic Serous Cystadenoma (MaSCA); Mucinous Cystadenomas (MCA); Mucinous Cystic Neoplasm (MCN); Mean CT Number (MCTN); Mass-forming Chronic Pancreatitis (MFCP); Machine Learning (ML); Multilayer Perceptron (MLP); Multi-channel-Multiclassifier-Random Forest (MMRF); Minimum Redundancy and Maximum Relevancy (mRMR); Pancreatic Cystic Lesion (PCL); Pancreatic Cystic Neoplasm (PCN); Pancreatic Adenocarcinoma (PDAC); Neuroendocrine Tumor (NET) or Pancreatic Neuroendocrine Tumor (PNET); Pancreatic Intraepithelial Neoplasia (PanIN); Pancreatic Neuroendocrine Neoplasm (PanNEN); Region of Interest (ROI); Radiomics Quality Score (RQS); Serous Cystadenomas (SCA); Serous Cystic Neoplasm (SCN); Standard Deviation (SD); Solid Pseudopapillary Neoplasm (SPN); Standardized Uptake Value (SUV); Volume of Interest (VOI).
